# Less Invasive Surfactant Administration: A Review of Current Evidence of Clinical Outcomes With Beractant

**DOI:** 10.7759/cureus.30223

**Published:** 2022-10-12

**Authors:** Manuel Sanchez Luna, Kristina Unnebrink, Marisol Martinez-Tristani, Cristina Ramos Navarro

**Affiliations:** 1 Neonatology, Hospital General Universitario Gregorio Marañón, Instituto de Investigación Sanitaria Gregorio Marañón, Madrid, ESP; 2 Data and Statistical Sciences, AbbVie Deutschland GmbH & Co. KG, Ludwigshafen, DEU; 3 Global Medical Affairs, Pharmaceutical Research and Development, AbbVie Inc., Chicago, USA; 4 Division of Neonatology, Instituto de Investigación Sanitaria Gregorio Marañón, Complutense University of Madrid, Hospital General Universitario Gregorio Marañón, Madrid, ESP

**Keywords:** less invasive surfactant administration (lisa), surfactant deficiency, respiratory distress syndrome, preterm infants, mechanical ventilation, insure, bronchopulmonary dysplasia

## Abstract

Evidence supporting clinical recommendations or approval for less invasive surfactant administration (LISA) has primarily examined heterogeneous or small-volume (e.g., 1.25-2.5 mL/kg) animal-derived surfactant regimens. To address the evidence gap for larger-volume (e.g., 4-5 mL/kg) animal-derived surfactants, the aim of this review was to evaluate and summarize LISA literature for widely used larger-volume beractant. Surfactant treatment and the LISA technique were initially summarized. The available literature on beractant with LISA was thoroughly assessed and reviewed, including a recent systematic analysis, studies from regions where access or preferences may influence reliance on larger-volume surfactants, and investigations of short- and long-term outcomes. The available literature indicated improved short-term outcomes, including less need for mechanical ventilation, death, or bronchopulmonary dysplasia, and no negative long-term developmental outcomes when beractant was administered via LISA compared with older, more invasive techniques. The rates of short-term outcomes were similar to those previously observed in examinations of LISA with small-volume surfactants, including in populations reflecting very preterm infants. As uptake of LISA is expected to increase, future research directions for larger-volume surfactants include cost-effectiveness evaluations and robust examinations of repeat dosing and surfactant reflux to further inform clinical practice. This review provides a detailed assessment of the literature describing surfactant and LISA, with a focus on studies of beractant. Collectively, the available evidence supports the use of beractant with LISA based both on short-term and long-term outcomes relative to more invasive techniques and comparability of outcomes with small-volume surfactants and may be valuable in guiding clinical decision-making.

## Introduction and background

Respiratory distress syndrome (RDS) commonly affects premature infants born with surfactant deficiency resulting from inadequate lung development (typically <37 weeks gestational age [wGA]) and may affect up to 80% of extremely preterm infants (≤28 wGA) [1,2]. Early administration of exogenous surfactant plays a key role in RDS treatment by reducing pneumothorax and improving survival [2]. Natural, animal-derived preparations are recommended, and the most commonly used animal surfactants are bovine-derived beractant and porcine-derived poractant alfa [2-4]. The traditional surfactant administration method uses intubation and mechanical ventilation during the procedure and potentially even afterward if extubation is not possible. However, this method of surfactant administration can cause lung injury, increasing the risk of bronchopulmonary dysplasia (BPD) or chronic lung disease [5], and may have long-term consequences. For example, BPD is a chronic lung disease that can persist until childhood and lead to other lung problems later in life [6].

Consequently, surfactant administration techniques have evolved to avoid prolonged exposure to mechanical ventilation and reduce the risk of adverse outcomes such as BPD [2,5]. The intubation-surfactant-extubation (INSURE) aims to achieve this by allowing for early extubation (within one hour) and switching from mechanical ventilation to nasal continuous positive airway pressure (CPAP) in spontaneously breathing preterm infants [2]. INSURE is not without limitations; even short-exposure mechanical ventilation may cause lung damage, and early extubation with CPAP is often not possible [7]. As a result, the newer less invasive surfactant administration technique (LISA), also referred to as minimally invasive surfactant therapy (MIST), was developed [8].

Instead of an endotracheal tube, the LISA technique primarily uses a thin and soft catheter inserted into the trachea, permitting spontaneous breathing while receiving noninvasive respiratory support and avoiding the use of invasive and potentially harmful ventilation methods [9,10]. According to clinical guidelines, the LISA procedure is generally viewed as a highly specialized technique, with its uptake varying regionally [9,11,12]. Like any surfactant administration method, it is recommended that LISA is performed by an experienced practitioner with the ability to intubate and provide mechanical ventilation, if needed [2]. In a recent multicenter training study in Poland that sought to increase the implementation of LISA in neonatal intensive care units, practitioners initially rated LISA as “easy” or “very easy” in 61% of cases, with this rate improving to 76% over a 12-month training period [12]. This suggests that uptake and implementation of LISA can be successfully increased with adequate practitioner training.

The European Consensus Guidelines recommend treating infants with RDS early with an animal-derived surfactant and identify LISA as the optimal method of surfactant administration, provided that clinicians are experienced with the technique [2]. These recommendations are supported by evidence from recent studies and meta-analyses suggesting that the use of LISA improves outcomes compared with other techniques such as INSURE [13-18]. However, this evidence, as well as available evidence supporting approval of LISA in several countries, has primarily included studies that focused on LISA but used heterogeneous surfactant regimens with different surfactant types, dosing, and animal source [15,18-20] or were specific to small-volume animal-derived surfactant (e.g., dose volume of 1.25-2.5 mL/kg) [18,19]. Although the initial study evaluating surfactant administration via LISA used the larger volume bovine-derived surfactant, beractant [8], outcomes following LISA with larger volume surfactants (e.g., dose volume of 4-5 mL/kg) are not as thoroughly studied, and randomized controlled trials are limited. As a result, scientific literature describing the outcomes of beractant with LISA is sparse, despite the status of beractant as a commonly used surfactant and LISA as the recommended administration method.

The aim of the current literature review is to address this evidence gap by aggregating and evaluating available clinical evidence from published studies of beractant with LISA to determine whether the benefits of LISA are observed with larger volume surfactants. Here, we review beractant’s clinical characteristics and its outcomes (not specific to LISA), in the context of another commonly used animal-derived surfactant. We also summarize available clinical evidence supporting the use of LISA over more invasive techniques and address the significant knowledge gap specific to LISA with larger volume surfactants by presenting a meta-analysis of key clinical outcomes for beractant administration with LISA (versus more invasive techniques) and reviewing other available literature describing LISA outcomes with larger volume surfactants. Lastly, we review additional evidence gaps and considerations related to LISA with larger volume surfactants and address likely future developments in the field. The authors hope that their systematic assessment of available evidence will help to inform clinical practice and policy decisions regarding surfactant administration in neonates with RDS.

## Review

Beractant treatment for RDS

Beractant is a natural bovine-lung extract with a phospholipid concentration of 25 mg/mL [21] and is administered in a dose of 100 mg phospholipids per kilogram of birthweight in a volume of 4 mL per kilogram of birthweight [2,21]. The amount of surfactant phospholipid delivered per dose is comparable to the normal phospholipid content of endogenous surfactant in at-term infants [22]. First approved by the US Food and Drug Administration (FDA) in 1991 and with more than three decades of real-world use, beractant is widely used for the treatment of RDS in preterm infants, including via LISA [2,3], and is included in the World Health Organization Model Lists of Essential Medicines [23]. Some clinical settings rely predominantly on a bovine-derived beractant due to cultural preferences and/or limited access to other options.

Beractant and another commonly used surfactant, poractant alfa, differ in phospholipid concentration and consequently dosing characteristics. Poractant alfa is a porcine lung extract with a phospholipid concentration of 74-76 mg/mL administered in a dose of 100 mg/kg (in a volume of 1.25 mL/kg) or in a dose of 200 mg/kg (in a volume of 2.5 mL/kg) [2,4]. The recommended initial dose, 200 mg/kg, is indicated for early surfactant treatment and has been associated with better outcomes (i.e., lower mortality and need for oxygenation) compared to 100 mg/kg [2,24], which may not be adequately or uniformly distributed inside the lungs due to its smaller dose volume [4,25-30]. Although pulmonary surfactant consists of 90% lipids, the remaining 10% is protein; both beractant and poractant alfa contain the SP-B and SP-C pulmonary surfactant proteins but lack the SP-D and SP-A types [31]. In addition to surface tension-lowering surfactant properties, SP-D and SP-A proteins play a key role in alveolar immunity and pulmonary inflammation [31], and supplementation of animal-derived surfactants with recombinant forms of these proteins has been postulated to play a potential role in the future treatment of RDS by further improving treatment outcomes [31].

Previous systematic evaluations of randomized controlled trials and real-world evidence have shown comparable outcomes for beractant and other commonly used animal-derived surfactants [4,32-34], although these studies did not examine outcomes by the method of administration (e.g., LISA vs INSURE or standard of care). A recent systematic review and meta-analysis of randomized controlled trials (RCTs), published in 2020, demonstrated beractant and poractant alfa treatment as having comparable rates of death, BPD, pneumothorax, and air leak syndrome [4]. Both surfactants were previously observed to have similar mortality rates in a 2015 network meta-analysis of RCTs [34] and similar rates of BPD, pneumothorax, and air leak syndrome in a 2015 Cochrane meta-analysis of RCTs [35]. Further, the 2015 Cochrane meta-analysis found no significant difference in one-month mortality rates, and evidence suggesting a potential difference in mortality rate prior to hospital discharge between beractant and poractant alfa 200 mg/kg [35] was not confirmed in the other two systematic evaluations investigating a larger body of literature [4,34]. This evidence of comparable outcomes with beractant and poractant alfa administered according to the US label (200 mg/kg) is further supported by evidence from use in clinical practice with the 2020 systematic review of four real-world studies (n = 66,112) observing similar rates of death, BPD, and air leak syndrome [4] and a recent retrospective study (n = 200) reporting similar mortality before discharge [32]. However, as mentioned earlier, these systematic evaluations did not examine the outcomes based on administration mode and particularly based on the LISA method.

LISA: Past clinical evidence and current research gap

The LISA technique for surfactant administration employs a small catheter placed in the trachea under direct laryngoscopy, eliminating the need for an endotracheal tube and intubation, which are inherent in the INSURE or standard-of-care techniques. This guideline-recommended technique minimizes the need for mechanical ventilation, which is required during intubation, and can cause lung injury and increase the risk of BPD, with potentially lasting consequences [2,5,6].

Systematic literature reviews and meta-analyses comparing the LISA technique with mechanical ventilation, INSURE, or nasal CPAP alone have shown that LISA is associated with significantly improved outcomes for BPD or death and a reduced need for mechanical ventilation within 72 hours of birth [15,18-20,36,37]. Failure of LISA was associated with younger gestational age (<28 weeks) and/or lack of prenatal steroids rather than the procedure itself or the surfactant used [29]. In terms of long-term outcomes, an early study suggested that infants treated with surfactant without intubation had no significant difference in neurodevelopment by the time they reached school age, compared with a historical cohort [38]. Two studies also found no differences in two-year clinical outcomes, including physical development (e.g., weight and height), neurodevelopment, psychomotor development, or respiratory outcomes in infants who received LISA versus standard methods (including INSURE or mechanical ventilation) [39,40]. Similarly, a third study showed no worsening of neurological outcomes in infants who received LISA after three years of follow-up [41]. Other investigations have reported better long-term outcomes with LISA versus INSURE, including increased survival without BPD and better psychomotor development scores [42,43].

A major gap in the body of evidence regarding the LISA method was the inclusion of multiple different surfactants that varied in sources and dose volumes [44,45], thus introducing potential confounding that may limit the generalizability of results to a specific surfactant in clinical practice. Studies that examined the clinical outcomes of LISA using a specific surfactant are generally limited to the small-volume, porcine-derived surfactant poractant alfa [18,19], while studies describing the outcomes after beractant treatment with LISA remain sparse, despite the fact that the first proof of concept study of the LISA technique was conducted with beractant and demonstrated the feasibility of this method [8]. Clinicians may often not have a choice of animal-derived surfactant available to them at their institution, but having necessary evidence supporting the clinical use of a given surfactant and route of administration can build confidence and better inform practice. Finally, some preclinical evidence points toward the need to demonstrate LISA effectiveness for bovine surfactants [46].

Beractant and LISA: an evidence update

Systematic Evaluation of Mechanical Ventilation, BPD, and Death

A comprehensive, systematic evaluation of beractant outcomes with LISA was recently presented at the Pediatric Academic Societies (PAS) 2021 meeting [47]. This review and meta-analysis examined beractant with LISA versus INSURE or standard of care in preterm infants with RDS by integrating original-research studies published between January 2007 and October 2020 and identified in BIOSIS Previews, Derwent Drug File, Embase, International Pharmaceutical Abstracts, MEDLINE, and SciSearch databases. Four key outcomes were extracted, namely, mechanical ventilation within 72 hours, BPD, death, and a composite outcome of BPD or death.

Among the seven studies included in the analysis (Table 1), two were RCTs [48,49], two were retrospective analyses [50,51], and three included comparisons with a historical control group [8,52,53]. Four of the included studies compared LISA versus INSURE [8,48,52,53], two compared LISA versus the standard of care (surfactant given after intubation [49] or intubation with surfactant followed by mechanical ventilation [51]), and one compared LISA versus INSURE as well as LISA versus conventional mechanical ventilation (analyzed as the standard of care in this analysis) [50]. Key inclusion/exclusion criteria and characteristics of the patient populations included in the seven studies are summarized in Table 1. Baseline characteristics did not vary significantly between the groups (LISA versus INSURE or standard of care). The number of patients in each study ranged from 45 to 267, and the proportion of males ranged from 40% to 71%. Gestational age (GA) inclusion criteria and mean GA ranged from 23 to 36 weeks and from 25.3 to 33.9 weeks, respectively. Overall, four studies examined study populations that reflect very preterm infants, based on the mean GA of 28-30 weeks and mean birth weight of 672-1240 g. One of these included a birth weight inclusion criterion of <1500 g (mean birth weight, 1141-1240 g; mean GA, 29 weeks) [51], and the other three studies included an upper limit for GA of 27-34 weeks (mean birth weight, 672-1232 g; mean GA, 25-31 weeks) [8,52,53]. The use of prenatal steroids ranged from 38% to 90% in included studies, likely reflecting the individual needs and practice preferences. Beractant was administered in all studies at a dosage consistent with the US product label (4 mL/kg of patient body weight, corresponding to 100 mg/kg; up to four doses can be administered every six hours within the first 48 hours of life) [21].

**Table 1:** Publications included in the Pediatric Academic Societies (PAS) 2021 systematic evaluation INSURE: Intubation, surfactant, then extubation; LISA, Less invasive surfactant administration; MV: Mechanical ventilation; N/A: Not available; nCPAP: Nasal continuous positive airway pressure; RDS: Respiratory distress syndrome; SOC: Standard of care; wGA: Weeks of gestational age; BPD: Bronchopulmonary dysplasia. ^a^24 randomized, 23 treated. ^b^Total N = 269; however, for two infants, two different methods of administration were used. The full analysis set comprised 267 infants. ^c^The study reported combined data for the full analysis set (i.e., all three treatment arms were combined).

Study	Key Criteria	Population	Patient Characteristics	Outcomes			
				BPD	Death	BPD or Death	MV
Kribs et al., 2007 [8], observational/historical control, location: Germany	23 to 27 wGA; treated with nCPAP immediately after birth for RDS; F_i_O_2_ ≥ 0.4 to reach SpO_2_ between 85% to 93%, or moderate to severe dyspnea at age ≥ 30 min	LISA (nCPAP + 100 mg/kg beractant), n = 29; Historical control (treated with early nCPAP and intubation for rescue surfactant therapy: INSURE), n = 34	Mean birth weight, LISA: 672 g; control: 716 g; Mean wGA, LISA: 25.4; control: 25.3; Male, LISA: 59%; control: 65%; Prenatal steroids, LISA: 90%; control: 85%	N/A	LISA: n = 2 (7%); Control: n = 12 (35%)	N/A	LISA: n = 10 (34%); Control: n = 26 (76%)
Ramos-Navarro 2016 et al., [52], prospective, open-label, nonrandomized/historical control, location: Spain	<32 wGA; spontaneous breathing on nCPAP during first three days of life; F_i_O_2_ > 30% (SpO_2_ 90% to 95%): surfactant first dose; F_i_O_2 _> 40% (SpO_2_ 90% to 95%): surfactant second dose	LISA (nCPAP + 100 mg/kg beractant), n = 30; Historical control (standard management with beractant after endotracheal intubation: INSURE), n = 30	Mean birth weight, LISA: 1058 g; control: 1232 g; Mean wGA, LISA: 28.4; control: 29.1 Male, LISA: 40%; control: 60%; Prenatal steroids, LISA: 73%; control: 70%	N/A	N/A	LISA: n = 8 (27%); Control: n = 9 (30%)	LISA: n = 13 (43%); Control: n = 22 (73%)
Olivier 2017 et al., [49], multicenter, randomized control, location: Canada	32 to 36 wGA; RDS within 24 hours of birth; F_i_O_2_ 35% and nCPAP support of 6 cm of H_2_O to maintain saturation ≥ 90%; no significant congenital malformations; no intubation or pneumothorax before enrollment	LISA (nCPAP + 100 mg/kg beractant), n = 24^a^; Control (standard management; surfactant was given only after intubation at physician’s discretion), n = 21	Mean birth weight, LISA: 2157 g; control: 2277 g; Mean wGA, LISA: 34.0; control: 33.9 Male, LISA: 42%; control: 71%; Prenatal steroids, LISA: 67%; control: 52%	N/A	N/A	N/A	LISA, n = 7 (29%); Control, n = 18 (86%)
Tomar et al., 2017 [53], single- center, prospective observational/historical control, location: India	>24 to <34 wGA; RDS due to surfactant deficiency with the need for supplemental oxygen with F_i_O_2_ > 0.4 to maintain SpO_2_ 85% to 92% in the first two hours after birth; no major congenital malformations; no resuscitation with intubation at delivery	LISA (nCPAP + 100 mg/kg beractant), n = 64; Control (intubation followed by surfactant: INSURE), n = 68	Mean birth weight, LISA: 1085 g; control: 1120 g; Mean wGA, LISA: 30.3; control, 30.6; Male, LISA: 47%; control: 51% Prenatal steroids, LISA: 73%; control, 65%	LISA, n = 1 (1.6%); Control, n = 4 (6%)	LISA, n = 2 (3%); Control, n = 3 (4%)	N/A	LISA, n = 13 (20%); Control, n = 18 (26%)
Halim et al., 2019 [48], single- center, randomized, location: Pakistan	<34 wGA with RDS; F_i_O_2_ adjusted to maintain SpO_2_ 88% to 92%; surfactant is given if F_i_O_2_ > 0.4 during the first 12 hours after birth; no congenital malformations; no intubation for resuscitation at birth	LISA (nCPAP + 100 mg/kg beractant), n = 50; Control (intubation and surfactant administration: INSURE), n = 50	Median birth weight, LISA: 1300 g; control: 1400 g; Male, LISA: 56%; control: 62% Prenatal steroids; LISA: 76%; control: 60%	N/A	LISA, n = 19 (38%); Control, n = 28 (56%)	N/A	LISA, n = 15 (30%); Control, n = 30 (60%)
Prutkin et al., 2019 [50], multicenter, retrospective, location: Russia	Treatment of RDS with beractant within the first 12 hours after birth; no major congenital malformations or chromosomal abnormalities; no receipt of other surfactants as mono or combined therapy	LISA, n = 60 INSURE, n = 107; SOC (conventional MV), n = 102^b^	Mean birth weight^c^: 1865 g; Mean wGA^c^: 32.0; Male^c^: 56% Prenatal; Steroids^c^: 38%	LISA, n = 5 (8%); INSURE, n = 8 (8%); SOC, n = 8 (8%)	N/A	N/A	N/A
Wang et al., 2020 [51], retrospective analysis, location: Taiwan	<32 wGA with signs of RDS despite nCPAP; birthweight <1500 g; F_i_O_2_ ≥ 0.4 to maintain SpO_2_ > 90%; no congenital malformations; no intubation immediately after birth	LISA (nCPAP + 100 mg/kg beractant), n = 24; Control (intubation, surfactant replacement therapy, followed by MV), n = 29	Mean birth weight, LISA: 1240 g; control: 1141 g; Mean wGA, LISA: 29.4; control: 28.7; Male, LISA: 58%; control: 59%	LISA, n = 5 (21%); Control, n = 13 (45%)	LISA, n = 0; Control, n = 1 (3%)	LISA, n = 5 (21%); Control, n = 14 (48%)	LISA, n = 2 (8%); Control, n = 29 (100%)

**Figure 1:**
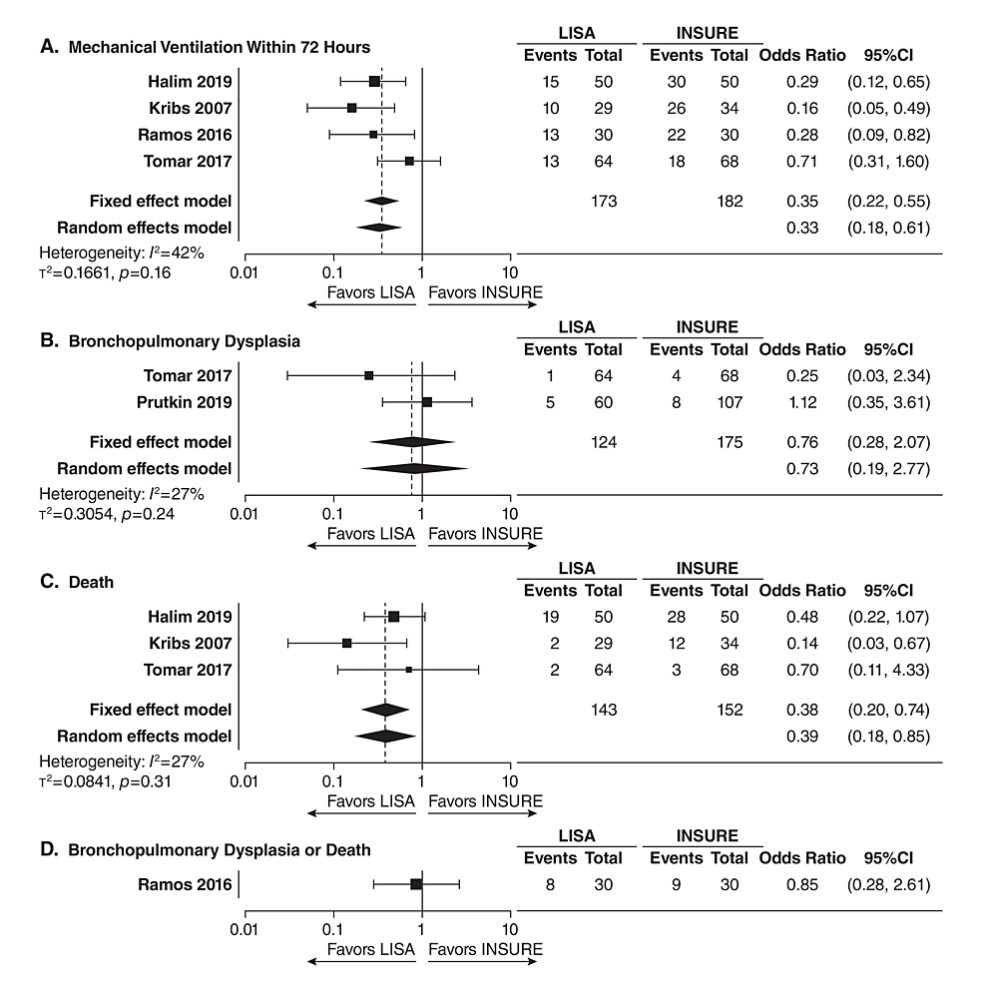
Comparison of LISA and INSURE treatment outcomes for (A) the need of mechanical ventilation within 72 hours, (B) bronchopulmonary dysplasia, (C) death, and (D) bronchopulmonary dysplasia or death Treatment effects are presented as odds ratios and 95% CIs. The dashed lines indicate the odds ratio for the fixed effects model. LISA: Less invasive surfactant administration; INSURE: Intubation, surfactant, then extubation.

**Figure 2:**
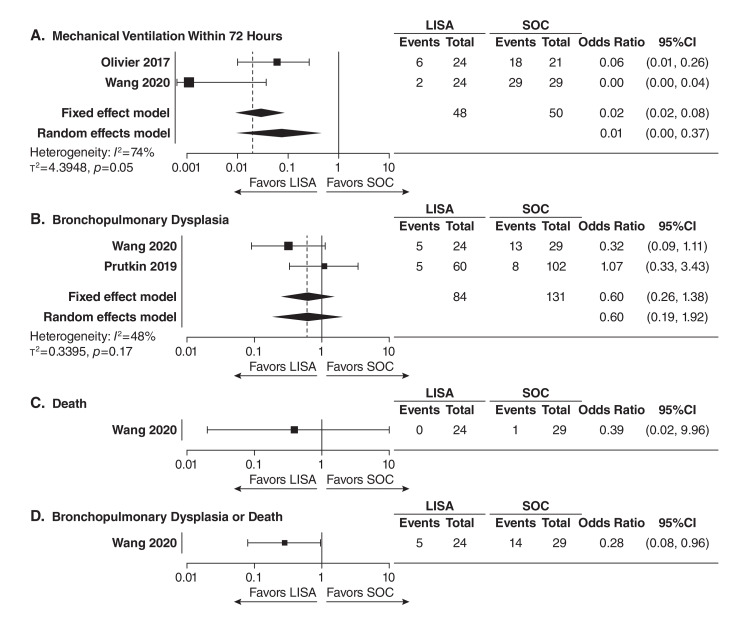
Comparison of LISA and standard of care treatment outcomes for (A) the need of mechanical ventilation within 72 hours, (B) bronchopulmonary dysplasia, (C) death, and (D) bronchopulmonary dysplasia or death Treatment effects are presented as odds ratios and 95% CIs. The dashed lines indicate the odds ratio for the fixed effects model. LISA: Less invasive surfactant administration; SOC: Standard of care.

**Figure 3:**
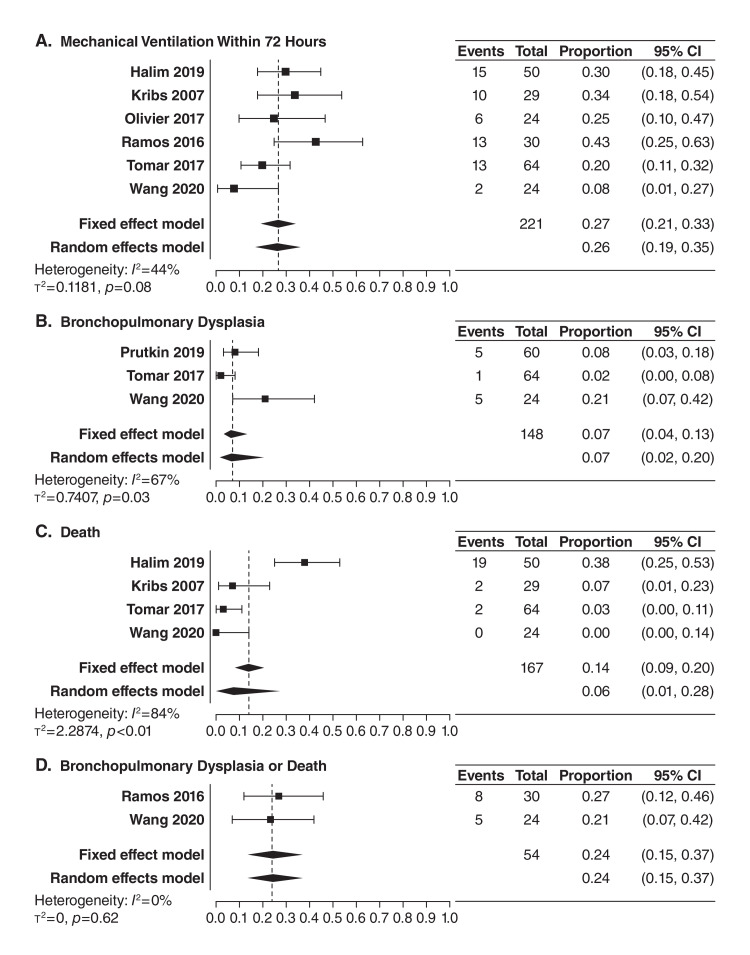
Event rates for LISA treatment for the need of mechanical ventilation within 72 hours (A), bronchopulmonary dysplasia (B), death (C), and bronchopulmonary dysplasia or death (D) Treatment effects are presented as the proportion of events in the total LISA treatment group. The dashed lines indicate the proportion for the fixed effects model. LISA: Less invasive surfactant administration.

Overall, none of the four outcomes examined favored INSURE or standard of care compared to LISA in this systematic evaluation, and the results of fixed effects and random effect modeling were comparable for the four extracted outcomes [47]. The results of this meta-analysis are described in detail here. For the treatment effect of LISA compared with INSURE (Figure 1, Panels A-D), the meta-analysis generally favored administration of beractant with the LISA method, with infants being 65% less likely to require mechanical ventilation within 72 hours (OR 0.35, 95% CI 0.22-0.55; four studies) and 62% less likely to die (OR 0.38, 95% CI 0.20-0.74; three studies) compared with INSURE. For the treatment effect of LISA compared with standard of care (Figure 2, Panels A-D), infants administered beractant with LISA were 98% less likely to require mechanical ventilation within 72 hours (OR 0.02, CI 95% 0.01-0.08; two studies) and 72% less likely to experience BPD or death (OR 0.28, CI 95% 0.08-0.96; one study) when compared with the standard-of-care methods. The following proportions (and 95% CI) of infants experiencing an outcome after beractant treatment with LISA were observed in a descriptive assessment of included studies (Figure 3, Panels A-D): mechanical ventilation within 72 hours, 27% (21%-33%; six studies); BPD, 7% (95% CI 4%-13%; three studies); death, 14% (95% CI 9%-20%; four studies); and BPD or death, 24% (95% CI 15%-37%; two studies). These data demonstrate a markedly lower rate of outcomes of interest with LISA versus other administration methods, especially for the statistically significant outcomes when considering the ORs of <0.5, and comparable with those observed in a prior meta-analysis of LISA using various surfactants [18,19]. When inspecting the four included studies that better reflected very preterm patients (Table 1) [8,51-53], only rates of mechanical ventilation within 72 hours (8%-43%) and BPD (2%-21%), but not death (3%-7%) or the composite outcome of death or BPD (21%-27%), had upper range limits falling outside of the upper limits of 95% CIs observed in the descriptive assessment of the entire study population. Together, the findings of this systematic evaluation of the aggregated evidence demonstrated that the LISA method, compared with INSURE or standard of care administration, was associated with improved key clinical outcomes in preterm infants with RDS treated with beractant. Low outcome rates were observed, including in very preterm infants, providing confirmatory evidence supporting the beractant use with LISA as recommended in current guidelines and some product regional labeling.

Additional Studies of Mechanical Ventilation, BPD, and Death

In addition to the studies included in the above systematic evaluation [47], several investigations from various countries throughout Asia, a region where access and cultural preferences may lead to a greater reliance on larger-volume bovine-derived surfactants, further confirm positive outcomes after beractant treatment with LISA. Two single-center observational studies conducted in Turkey compared infants (<35 wGA) treated with 100 mg/kg beractant or 200 mg/kg poractant administered with LISA and found no significant differences in the rates of mechanical ventilation [54], BPD [54,55], and death [54,55] between the two treatments, although these studies were limited by small sample sizes (n = 30-54) and unbalanced groups based on birthweight. A three-center RCT conducted in India compared the administration of a larger-volume bovine surfactant (Neosurf, 5 mL/kg, 135 mg phospholipids per kg) with LISA (n = 175) versus INSURE (n = 175) and found lower rates of mechanical ventilation in the first 72 hours (19% versus 40%) and BPD (3% versus 17%) in infants (≤34 wGA) treated with LISA [56] compared to INSURE, similar to previously discussed outcomes observed with beractant and LISA. These results further extend the body of evidence suggesting positive outcomes following the beractant treatment with LISA.

Long-Term Developmental Outcomes

One study (included in the systematic analysis previously discussed) examined long-term developmental outcomes in infants treated with beractant and LISA. This single-center retrospective chart review of 60 preterm infants (<32 wGA) on CPAP during the first three days of life observed no significant differences in the rates of acute respiratory failure admissions, hearing loss defects, and neurological disorders between the infants treated with beractant and LISA (n = 30) versus INSURE (n = 30) during a long-term follow-up of up to 24 months of postmenstrual age [40]. Taken together, the available literature indicates improved short-term outcomes, and no negative impact on long-term outcomes assessed when beractant (a larger-volume surfactant) is administered via LISA compared with older, more invasive techniques.

Evidence gaps and future directions

A cost-effectiveness evaluation of preterm infants with RDS observed a shorter length of NICU stay and lower total newborn intensive care unit (NICU) costs for beractant compared to other commonly used animal-derived surfactants at 209 US hospitals (n = 13,240) [57]. However, this study examined heterogeneous administration methods, thus potentially not reflecting LISA-specific costs. The RCT from India discussed above observed a shorter median hospital stay for a larger-volume bovine surfactant (Neosurf, 5 mL/kg, 135 mg phospholipids per kg) administered with LISA versus INSURE (11 versus 28 days) [56], suggesting cost savings with the LISA technique, but a comprehensive health-economic evaluation is needed to confirm these findings.

Additional studies are also needed to more robustly examine repeat dosing with LISA, which typically involves tracheal visualization to guide the surfactant administration and may consequently impact the clinical outcomes, especially when a repeat surfactant administration is needed. In a historical cohort study of 324 infants, patients receiving surfactant with LISA required more doses of surfactant but ultimately needed less mechanical ventilation than those receiving surfactant with intubation and had other improved outcomes [37]. With a larger-volume bovine-derived surfactant, less need for repeat dosing could be potentially expected since, as mentioned earlier, a larger volume may lead to a better surfactant distribution in the lungs [4,25-30]. Many key studies of beractant with LISA permitted repeat dosing based on oxygen requirements, and in four studies that reported a second dose, the proportion of infants requiring a second dose ranged from 17% to 38% with LISA compared to 22%-31% with INSURE or standard of care [49,51-53]. However, the studies that reported the number of patients who received a second dose did not report a correlation with outcomes such as mechanical ventilation, and thus, the effect of multiple doses was not assessed, warranting additional research. Future studies comparing repeat dosing between different animal-derived surfactants administered with LISA should strive to examine repeat dosing over an adequate time period encompassing dosing intervals for different surfactant types (i.e., ≥12 hours) and also to assess the total cumulative dose of phospholipids received. Furthermore, repeat dosing and total cumulative dose should be examined in association with clinical outcomes, since in the context of LISA, the key concern is not only the costs of repeat dosing but also the potential influence of LISA-specific techniques (e.g., tracheal visualization) on RDS outcomes.

Surfactant reflux is another outcome warranting more robust research since some groups have observed increased instances of reflux or bradycardia with LISA compared to INSURE when examining various types of surfactants [18,19,36]. Others have noted their expectation to observe higher rates of reflux with larger-volume surfactants when utilizing LISA [24]; however, studies of the larger-volume surfactants such as bovine lipid extract surfactant (BLES) and Neosurf (5 mL/kg, 135 mg phospholipids per kg) administered with LISA have generated inconsistent data regarding reflux and reflux-related outcomes [45,56]. Results from chart reviews examining reflux rates with larger-volume beractant versus small-volume poractant administered via LISA have also been inconsistent and were limited, in part, by the small sample size [54,58]. Additional evidence assessing reflux with LISA for different surfactants could help inform efforts for practitioner education and training and should, therefore, also describe relevant LISA administration techniques utilized to proactively minimize this outcome. For instance, a proactive technique was used in one of these chart reviews, where the stomach was aspirated before LISA and the surfactant was dispersed during the procedure via air flush until there was no regurgitation evident. No patients receiving either beractant or poractant experienced reflux, potentially due to the use of this technique [58]. So, future evaluations of reflux and bradycardia should consider potential intercenter differences, by describing and adjusting for relevant techniques in their analysis.

Finally, as the LISA technique has become more widely used in clinical practice [2,12,45], the potential of a truly noninvasive method of surfactant administration, i.e., nebulization through aerosols, is currently being investigated [2,24]. Some experts have highlighted this technique as a promising future direction that could help further improve the clinical outcomes of RDS due to its noninvasive nature [2,24]. As the earlier clinical studies of jet nebulizers observed no added benefits, surfactant nebulization has undergone various improvements with newer administration techniques and nebulizers being able to deliver higher doses of surfactants [2,24]. Clinical research examining the feasibility, safety, and efficacy is underway for this noninvasive method of surfactant administration, including for beractant [59].

## Conclusions

Today, there is good evidence supporting the recommendation of the LISA technique as an alternative to standard or INSURE methods of surfactant administration. As most of the recent studies analyzing the potential benefits of the LISA have been done with small-volume surfactants (e.g., 1.25-2.5 mL/kg), some doubts could exist when LISA is performed with larger surfactant volumes. Therefore, the main objective of this review was to summarize the evidence for the effectiveness and safety of LISA administration with a larger-volume surfactant (e.g., 4-5 mL/kg), compared to its administration with INSURE or standard-of-care techniques. When considered collectively, the available evidence summarized in this review supports the use of LISA with the larger-volume beractant.

The first systematic evaluation of studies investigating the key clinical outcomes following beractant administration using the LISA technique clearly demonstrated, despite the differences in source studies, better outcomes with beractant administered via LISA vs INSURE or standard of care. Further evidence from additional studies conducted in various Asian countries further confirms the results of the systematic evaluation. The results of this review extend from previous reports and support the clinical use of beractant administered with LISA by demonstrating that this approach results in fewer adverse outcomes for preterm infants with RDS when compared with INSURE or standard of care. Importantly, the bovine-derived surfactant is included in the World Health Organization Model Lists of Essential Medicines for neonates. By focusing on this frequently used surfactant therapy, this review provides a summary of administration methods without potential confounding factors introduced by multiple surfactants from different animal sources and different doses of phospholipids. While the LISA method may eventually become replaced by surfactant nebulization or other noninvasive techniques, it is being increasingly adopted in current practice, in accordance with European treatment guidelines, with training resulting in good proficiency and comfort level among trained practitioners. We hope that the findings of this review will help guide clinical management decisions and improve patient outcomes in preterm infants with RDS and that they will be particularly informative to practitioners in regions where beractant and other larger-volume surfactants are predominantly used and access to other surfactants may be limited.
